# Life Expectancy at Birth for People with Serious Mental Illness and Other Major Disorders from a Secondary Mental Health Care Case Register in London

**DOI:** 10.1371/journal.pone.0019590

**Published:** 2011-05-18

**Authors:** Chin-Kuo Chang, Richard D. Hayes, Gayan Perera, Mathew T. M. Broadbent, Andrea C. Fernandes, William E. Lee, Mathew Hotopf, Robert Stewart

**Affiliations:** 1 King's College London (Institute of Psychiatry), London, United Kingdom; 2 South London and Maudsley NHS Foundation Trust, London, United Kingdom; 3 King's College London, Academic Dept Psychological Medicine, Institute of Psychiatry, London, United Kingdom; The University of Queensland, Australia

## Abstract

**Objective:**

Despite improving healthcare, the gap in mortality between people with serious mental illness (SMI) and general population persists, especially for younger age groups. The electronic database from a large and comprehensive secondary mental healthcare provider in London was utilized to assess the impact of SMI diagnoses on life expectancy at birth.

**Method:**

People who were diagnosed with SMI (schizophrenia, schizoaffective disorder, bipolar disorder), substance use disorder, and depressive episode/disorder before the end of 2009 and under active review by the South London and Maudsley NHS Foundation Trust (SLAM) in southeast London during 2007–09 comprised the sample, retrieved by the SLAM Case Register Interactive Search (CRIS) system. We estimated life expectancy at birth for people with SMI and each diagnosis, from national mortality returns between 2007–09, using a life table method.

**Results:**

A total of 31,719 eligible people, aged 15 years or older, with SMI were analyzed. Among them, 1,370 died during 2007–09. Compared to national figures, all disorders were associated with substantially lower life expectancy: 8.0 to 14.6 life years lost for men and 9.8 to 17.5 life years lost for women. Highest reductions were found for men with schizophrenia (14.6 years lost) and women with schizoaffective disorders (17.5 years lost).

**Conclusion:**

The impact of serious mental illness on life expectancy is marked and generally higher than similarly calculated impacts of well-recognised adverse exposures such as smoking, diabetes and obesity. Strategies to identify and prevent causes of premature death are urgently required.

## Introduction

Concerns about premature mortality among people with mental disorders have been increasing [1]–[6]. Higher general and specific causes of mortality in all or specific age groups have been identified for people with serious mental illnesses (SMI, which might include schizophrenia, schizoaffective disorder, bipolar disorder, and depressive psychosis) in a range of settings [1], [2], [7]–[11], although most report relative risks or rates of mortality rather than life expectancy – which is arguably a more important index for shaping policy since it takes premature mortality into account and has been widely used for other risk exposures such as smoking, diabetes, obesity, ethnicity and socioeconomic status [12]–[17]. Of the few studies to measure this outcome, clients with SMI from public mental health agencies in eight US states from 1997 to 2000 were found to have lower life expectancies of 13 to more than 30 years compared to the general population (depending on the year and state) [2], and a loss of 8.8 life years (14.1 years for men and 5.7 years for women) was estimated in a comparison between people treated for SMI and the general population in Massachusetts, US [1]. A Swedish study using a nationwide hospital discharge registry reported substantial gaps in life expectancy at age 30 for main mental disorder categories compared to the general population, particularly for functional psychosis other than schizophrenia / affective psychosis (15.9 years lost), substance abuse (15.6 years lost), and organic psychosis (14.8 years lost) among men and organic psychosis (22.6 years lost), mental retardation (14.7 years lost), and substance abuse (18.8 years lost) among women [18]. Preliminary strategies for preventing premature deaths among people with SMI have been suggested, emphasizing the management of suicide risk and physical illness, minimum polypharmacy, and improvement of accessibility to physical health care [19].

Despite healthcare improvements, there has been little evidence of benefit on life expectancy in people with SMI [1], [20], [21], and further research is urgently required [22]. We reported excess mortality for people with diagnoses of SMI, as well as for depressive and substance use disorders, drawing on data from the South London and Maudsley NHS Foundation Trust Biomedical Research Centre (SLAM BRC) Case Register, which covers comprehensive secondary mental healthcare provision to a large geographically defined community, by standardized mortality ratios (SMRs) [23]. Nonetheless, equal weighting is used for each age group in the calculation of SMRs, which is insufficient for highlighting the impact of premature mortality among younger age groups in the study cohort.

## Methods

### Ethics Statement

The study was approved as an anonymized dataset for secondary analysis by the Oxfordshire Research Ethics Committee C (reference 08/H0606/71).

### Study Setting

Under the National Health Service (NHS) system in the UK, there is universal state provision of healthcare, and mental health Trusts have a close to 100% monopoly for service provision to defined geographic catchment areas. The South London and Maudsley NHS Foundation Trust (SLAM) is Europe's largest provider of secondary mental healthcare, serving four boroughs in southeast London (urban and suburban areas) with a geographic catchment of approximately 1.2 million residents and provision of all aspects of secondary mental healthcare to all age groups including inpatient, community, general hospital liaison and forensic services. Since 2006, fully electronic clinical records have been implemented in all SLAM services. The Case Register Interactive Search (CRIS) system supported by the NIHR Specialist Biomedical Research Centre for Mental Health, was developed in 2008 to allow searching and retrieval of anonymized full records for over 165,000 cases currently represented in the system [24].

### Mortality Detection

Detailed methods describing how the cohort of SLAM patients with serious mental illness were identified for this study are described elsewhere [23]. In brief, routine mortality identification is performed on a monthly basis by SLAM through the linkage of all NHS numbers (unique identifiers) on the case records database (including both active and inactive cases) to nationwide tracing provided by the NHS Care Records Service. All-cause mortality was investigated over a three-year period from Jan 1, 2007 to Dec 31, 2009. Mental disorder diagnoses are assigned routinely by SLAM clinical staff using the International Classification of Diseases (ICD) coding system. Service users who contacted SLAM before the end of 2009 (i.e. referral, discharge, or case note entry) and received at least one diagnosis of SMI (schizophrenia (ICD10 - F20), schizoaffective disorders (F25), and bipolar affective disorder (F31)), substance use disorder (F10 to F19), depressive episode (F32), or recurrent depressive disorder (F33) prior to 2010 were included in the cohort. Five-year age groups (0–4, 5–9, … 85–89, and 90+ years old) were defined according to age calculated at the mid-point of the follow-up period (i.e. Jul 1^st^, 2008). For each diagnosis, the size of population in 5-year age groups and counts of deaths in the same age groups were obtained by gender.

### Statistical Method

Life expectancy at birth is a demographic index which is a feature of overall mortality emphasizing the impact of deaths occurring in younger age groups. It is estimated from the age-specific mortality of a specific cohort over a given period of time using the life table method and is calculated from the accumulated person-years contributed by the entire cohort divided by the total population number at birth [25]. Methodological recommendations from the UK Office for National Statistics (ONS) have been published [26], based on which a series of reports on life expectancy at birth for sub-national areas of the United Kingdom were prepared. Using Microsoft Excel, the results for the SLAM cohorts were calculated using Chiang's method of abridged life tables in 5-year groups, which has been recommended for its application to relatively small populations allowing standard errors to be estimated [27]. In practice, since those under 15 years of age would be unlikely to receive an SMI diagnosis, we used the UK mortality rates for the under 15 year age groups in substitution [28]. We then combined this with the mortality associated with specific mental disorders. The differences in life expectancy at birth between diagnostic groups and those for the UK population in 2006–08 [29] were also calculated by gender. Multiple diagnostic coding was rare during the period of interest. However, disorder groups were analysed separately so that people with more than one disorder coding in the surveillance period would appear in the output for each disorder.

## Results

A total of 38,066 people were identified using CRIS with a primary diagnosis of schizophrenia, schizoaffective disorder, bipolar affective disorder, substance use disorder, depressive episode, or recurrent depressive disorder, recorded before the end of 2009. Among them, 5,902 subjects were not receiving active care from SLAM services at any point during the surveillance period and were thus excluded from analyses in order to increase the generalisability to other secondary mental healthcare samples (i.e. so that the findings could be taken as pertaining to a clearly defined sample). Those younger than 15 years old at the mid-point of 2008 or with a missing date of birth were also excluded (n = 445). Therefore, a total of 31,719 cases of interest with 1,370 deaths were identified in the study period. Of the sample, 1,680 (5.3%) had two diagnoses of interest before the end of 2009, 121 (0.4%) had three diagnoses and six (0.02%) had four diagnoses. The most common combinations of diagnoses applied on different occasions in the same individual were schizophrenia and schizoaffective disorders (n = 421), followed by substance use disorders and depressive disorders (n = 352).

**Figure 1:**
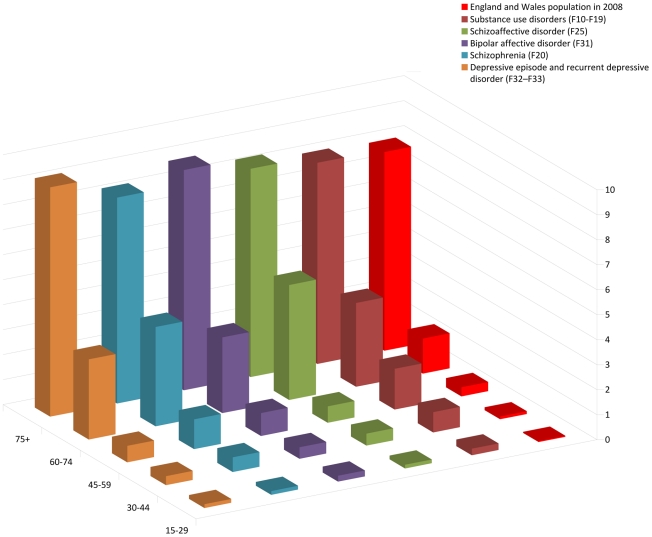
Annual mortality risk (%) by age groups and diagnoses of mental illness, compared to England and Wales population in 2008.

In the analyzed sample, 54.0% were male, and the mean (SD) ages were 43.1 (14.7) years in men and 45.1 (18.0) years in women. Cell sizes and crude mortality risks are displayed in Table 1. Three-year mortality rates increased substantially by age groups in both genders, and were higher for men in all age groups. Mortality was highest in men with depression and women with schizophrenia. Mortality by age group and diagnosis was further displayed in Figure 1.

**Table 1:** Mortality, demographic characteristics and serious mental illness diagnosis of SLAM in the period of 2007–09 (N = 31,719; death = 1,370)∧.

Variable	Number (number of deaths, %mortality)	
	Male (n = 17,113)	Female (n = 14,579)
Age groups		
15–29	3,202 (25, 0.78)	3,091 (11, 0.36)
30–44	7,410 (159, 2.15)	5,224 (65, 1.24)
45–59	4,351 (165, 3.79)	3,403 (95, 2.79)
60–74	1,473 (188, 12.76)	1,535 (127, 8.27)
75–89	621 (193, 31.08)	1,185 (258, 21.77)
90+	56 (27, 48.21)	141 (57, 40.43)
SMI diagnosis		
Schizophrenia (F20)	4,426 (196, 4.43)	2,592 (126, 4.86)
Schizoaffective disorders (F25)	652 (16, 2.45)	660 (28, 4.24)
Bipolar affective disorder (F31)	1,126 (43, 3.82)	1,573 (65, 4.13)
Substance use disorders (F10–F19)	7,654 (254, 3.32)	3,268 (94, 2.88)
Depressive episode and recurrent depressive disorder (F32–F33)	4,264 (284, 6.66)	7,417 (336, 4.53)

∧ Number of missing gender information = 27.

Table 2 summarized the calculated life expectancies at birth by gender for specific diagnoses and their differences from national norms. Life expectancies at birth for people with mental disorders ranged from 62.8 (schizophrenia) to 69.4 (schizoaffective disorders) years in men, and from 64.1 (schizoaffective disorders) to 74.4 (depressive disorders) years in women. All the life expectancies at birth for specific mental disorders were significantly different between genders based on their 95% confidence intervals. Wide disparities between genders were found for schizophrenia and schizoaffective disorders. Specifically, men with schizophrenia had 14.6 years reduced life expectancy compared to a reduction of 9.8 years for women; meanwhile, women with schizoaffective disorders had 17.5 years reduced life expectancy compared to a reduction of 8.0 years for men.

**Table 2:** Life expectancy at birth of people with specific mental disorders in the period of 2007–09 (N = 31,719).

Diagnosis	Male		Female	
	Life Expectancy (95% CI, number of deaths)	Difference from male UK population*	Life Expectancy (95% CI, number of deaths)	Difference from female UK population*
**Any Serious Mental Illness** ∧	64.5 (63.3–65.6, n = 243)	−12.9	69.9 (68.7–71.0, n = 203)	−11.8
Schizophrenia (F20)∧	62.8 (61.6–64.10, n = 196)	−14.6	71.9 (71.0–72.8, n = 126)	−9.8
Schizoaffective disorder (F25)∧	69.4 (68.3–70.5, n = 16)	−8.0	64.1 (60.9–67.2, n = 28)	−17.5
Bipolar affective disorder (F31)∧	67.3 (66.1–68.5, n = 43)	−10.1	70.4 (69.5–71.4, n = 65)	−11.2
**Substance use disorders (F10–F19)** ∧	63.9 (62.7–65.0, n = 254)	−13.6	66.9 (65.5–68.3, n = 94)	−14.8
**Depressive episode and recurrent depressive disorder (F32–F33)** ∧	66.8 (65.6–67.9, n = 284)	−10.6	74.4 (73.5–75.3, n = 336)	−7.2

*Life expectancy at birth 2006–08 in UK: Male = 77.4 years; Female = 81.6 years [27].

∧ Significant difference between genders.

## Discussion

Using a large secondary mental healthcare database in southeast London linked to national mortality returns, we confirmed substantially shortened life expectancies at birth for all serious mental disorder groups investigated compared to national norms. Largest reductions were found for men with schizophrenia, women with schizoaffective disorders, and both men and women with substance use disorders. In previous analyses of these cohorts, raised standardised mortality ratios (SMRs) had been described [23], although the picture was less dramatic because SMRs reflect less well the impact of premature mortality at earlier life stages. In a US study conducted by the Massachusetts Department of Mental Health from 1989 to 1994, service users with SMI were found to have lost 8.8 years of potential life (14.1 years for men and 5.7 for women) on average compared to the general population, a stronger impact for men and weaker impact for women compared to findings from our sample. However, SMI was not specifically defined in the Massachusetts study other than stating “evidence of serious dysfunction” as an inclusion criterion [1], which might explain the discrepancy. A Swedish analysis of national discharges from 1978–82 reported more complex data on life expectations at given ages. For the lowest age described (30 years), subsequent life expectancy associated with schizophrenia was 7.9 years lower in men and 9.5 years lower in women [18] – i.e. similar to the finding for women in our sample but less strong for men. Further international research is clearly required to compare the potential impacts of SMI on life expectancy in different settings since this may provide clues as to systems of care which may help to maximise this.

Mortality in SMI is recognised to be raised and underlying causes may be multiple. As well as our own findings, any psychiatric diagnosis was associated with a 65% higher than expected total mortality in a case register study in a British primary care cohort [30]. In the same setting, a three-fold elevated mortality was found for coronary heart disease in young adults with SMI, and a more than two-fold increased mortality of stroke in all age groups [8]. Long-term antipsychotic use and adverse lifestyle choices (e.g. obesity, smoking, poor diet, illicit drug use, and physical inactivity) are implicated in increased risk of cardiovascular events in these populations [3], [19], [20], [22], [31]–[33] and clearly need higher levels of consideration in order to improve health and survival, as well as the better-known risks of suicide and violent deaths. Because the causal pathways between mental disorder and premature mortality are multiple, and because life years lost as an outcome measures not only the effect of an exposure on risk of mortality but also its differential effect across the age range, it is difficult to interpret with certainty the gender variation in findings – i.e. the strongest effects of schizophrenia in men and schizoaffective disorder in women. Clearly the two disorders overlap substantially at a genetic and biological level. It is possible that the impact of co-occurring affective disorder is stronger in women due to specific biological or social vulnerability. However, against this, there was no difference in the impact of bipolar disorder between men and women and the impact of depressive episode/disorder was stronger in men. Alternative explanations may lie in the clinical practice of assigning diagnoses and gender differences here – for example, if there was preferential inclusion in women of severe personality disorders with transient psychotic episodes within this diagnostic group. Further research is clearly required to clarify this issue.

Life expectancy is a commonly used indicator for how longevity may be impaired by specific long-term exposures (e.g. smoking, obesity, ethnicity, and socioeconomic status) or chronic states of ill-health or risk (e.g. diabetes mellitus) [12], [13], [15]–[17], [34], and provides an alternative platform to discriminate the influences of different exposures for the purpose of highlighting premature mortality at younger ages in potentially vulnerable groups [25]. It is therefore primarily a measure of *impact* and should be seen as complementary to more common studies using measures of *effect*, such as SMR statistics. As a measure, life expectancy analyses provide an important means of communicating impact on survival to policy makers and lay audiences. This approach also allows the impact of different exposures to be compared in a way which cannot be inferred from SMR outputs. As a case in point, considering the impact of other well-recognised exposures and comparing these with the potential impact of SMI, in a Japanese study of life expectancy from age 40, smoking was the most important predictor of six lifestyle related exposures (smoking, alcohol consumption, walking duration, sleep duration, consumption of green leafy vegetables, and obesity), current smoking being associated with around four to five life years lost for both genders [12], [13]. Considering 2005 life expectancy levels in the USA (75.1 years for men and 80.3 years for women), current and previous smoking was associated with 2.5 years lower life expectancy for men and 1.8 years lower life expectancy for women [17]. For British doctors born in 1900–1930 and followed up for fifty years, continued smoking was associated with around 10 years life lost, compared to lifelong non-smokers [16], and in New Zealand Census records in 1996, the gap in life expectancy between current and never smokers was 7.6 years in men and 6.7 for women [15]. Diabetes was reported to reduce life expectancy at age 15 by 1.3 years for men and 2.0 years for women in Canada [14], and a BMI of 40–45 kg/m^2^ was associated with a 10 year reduction of life expectancy at age 35 compared to a BMI 22.5–25 kg/m^2^ [35]. The potential impact of having a serious mental illness (or a substance use disorder or a depressive episode requiring secondary care contact) on life expectancy is therefore at least comparable and at worse substantially higher than effects of individual well-recognised exposures, although clearly the mechanisms through which mental disorders are associated with premature mortality will include the effects of these individual risk factors (for example, smoking behaviour, risk of diabetes, etc.) as well as other factors (such as risk of suicide or accidents and direct effects of mental distress on cardiovascular risk). Excess mortality associated with mental disorders has been demonstrated to be predominantly due to ‘natural’ causes [3], [5], [30], [31], [36]–[38], although mental health service provision is often focused on preventing more rare outcomes of suicide and violent death [39]–[41]. If improving overall survival is to be considered as an alternative priority, much more efforts are clearly required to address the challenges of improving general health in people with mental disorders through medical services, socioeconomic support, and physical health promotion strategies [19].

Strengths of the study described include the large sample size and nationwide coverage of mortality, as well as the context of complete electronic clinical records. Potential limitations include the secondary healthcare setting; this should present no problem for high penetrance disorders, such as schizophrenia and bipolar disorder, where most cases will have received secondary care input. However, findings for substance use disorders and depressive disorders should be viewed with circumspection since those appearing on a secondary care register are likely to be an unrepresentative (and probably more severe) subset of community cases. Ultimately, the findings for life expectancy should be taken as referring to people with these mental disorders who had made contact with secondary mental health services within the given time period and are not necessarily applicable to all cases in a given community. Diagnostic categories overlapped, but only a minority (5.7%) of people had received more than one mental disorder diagnosis during the surveillance period. Nevertheless, comorbidity between SMI and substance use disorders should not be assumed to be captured as the source data reflected primary diagnoses rather than co-occurrence. Also we did not attempt to capture co-occurring or changing diagnoses which present potentially complex issues for analysis in a secondary care sample and which we felt were beyond the scope of this initial report. Although SLAM is a major academic as well as clinical centre, mental disorders on the Case Register are likely to be broadly generalizable to other urban settings in the UK (the SLAM catchment covers a range of residence areas from inner city to suburban settings but does not include any rural areas). Life expectancy comparisons of regional data to national norms may be problematic. However, in a sensitivity analysis of SMR data, the use of London comparators did not meaningfully change findings [23]. Also, from administrative data, life expectancy at birth for population of the four catchment area boroughs in 2007–09 (ranging from 76.3–79.5 years for males and 81.1–82.9 years for females), were not substantially different to national estimates [42].

Our results demonstrate the substantial impact of serious mental illness on life expectancy and highlight importance of developing strategies to prevent premature mortality among people with SMI and other major mental disorders. Although the prevention of suicides and violent deaths are likely to be an important component, other causes of excess mortality should not be ignored, including cardiovascular diseases, cancer, and diabetes [3], [4], [7], [8], [22], [31]. Physical health risk assessments and assertive intervention by primary and secondary medical services have been proposed and have resulted in improvements [20], [43]. Continued monitoring of mortality trends in people with mental disorders will be required to evaluate the impact of policy changes as they occur.
